# Evaluation of the Efficacy of Plasma Shower and 585 Nm Q‐Switched Laser in the Treatment of Mild to Moderate Acne Vulgaris

**DOI:** 10.1111/jocd.70876

**Published:** 2026-04-21

**Authors:** Celal Alperen Bakircioglu, Dursun Turkmen, Nihal Altunisik, Zekiye Kanat, Serpil Sener, Cemil Colak

**Affiliations:** ^1^ Inonu University, Faculty of Medicine Department of Dermatology Malatya Türkiye; ^2^ Inonu University, Faculty of Medicine Department of Biostatistics and Medical Informatics Malatya Türkiye

**Keywords:** 585 nm Q‐switch laser, acne vulgaris, cold atmospheric plasma, laser therapy, plasma shower

## Abstract

**Objective:**

The aim of this study is to comparatively evaluate the clinical efficacy, safety profiles, and effects on different clinical components of plasma shower and 585 nm Q‐Switch laser in the treatment of mild to moderate acne vulgaris.

**Materials and Methods:**

A total of 47 patients with mild to moderate acne vulgaris were prospectively enrolled and allocated to either plasma shower (*n* = 24) or 585 nm Q‐Switch laser (*n* = 23) treatment groups. Acne severity was assessed using the Global Acne Grading System (GAGS) at three time points: Baseline, week 2, and week 4. Repeated measures ANOVA and independent samples *t*‐test were used for statistical analysis.

**Results:**

An average improvement of 42.5% in GAGS scores was observed in the plasma shower group at the fourth week, while this rate was 32.2% in the 585 nm Q‐Switch laser group. This difference between the two groups was statistically significant (*p* = 0.007). Clinical observations revealed that the plasma shower provided faster and more pronounced regression in inflammatory papules and pustules, while the 585 nm Q‐Switch laser was more effective on post‐inflammatory erythema. Both methods were found to be safe and well‐tolerated by patients.

**Conclusion:**

Plasma shower appears to be more effective than the 585 nm Q‐Switch laser in the treatment of mild to moderate acne vulgaris, particularly in reducing active inflammatory lesions. The 585 nm Q‐Switch laser is a valuable treatment option for the removal of post‐inflammatory erythema. Clinicians may choose between these modalities or develop combined protocols based on the predominant clinical component of acne.

## 1 Introduction

Acne vulgaris is a chronic, inflammatory disease of the pilosebaceous unit. It is a significant dermatological problem that primarily affects the adolescent population but is also increasingly prevalent in adulthood [1, 2]. Its pathogenesis involves sebum hypersecretion, follicular hyperkeratosis, colonization by Cutibacterium acnes (C. acnes), and the subsequent development of immune‐mediated inflammatory processes [3]. This multifactorial structure explains the broad clinical spectrum of the disease and the variability in response to treatment.

Current epidemiological data indicate that acne vulgaris has a prevalence of approximately 9.4% worldwide [4]. Studies conducted in Turkey also confirm that acne is a common dermatological problem, particularly in young adults, which significantly reduces quality of life [5]. The incidence and severity of the disease are influenced by parameters such as genetic predisposition, hormonal factors, and environmental factors, and may also vary depending on geographical and ethnic origin [5, 6]. The cosmetic problems caused by acne can lead to psychosocial stress, loss of self‐confidence, and a decline in quality of life, particularly during adolescence, highlighting the need for effective and rapid treatment approaches [7].

Traditional medical treatments such as topical retinoids, antibiotics, benzoyl peroxide, and systemic agents constitute the first‐line options for the treatment of mild to moderate acne vulgaris [7]. However, limitations such as drug side effects, non‐compliance with treatment, increasing antibiotic resistance, and long treatment durations have led to a need for alternative and complementary treatment modalities [8]. In this context, laser and energy‐based devices and physical therapy methods have become an increasingly important area of research and application in recent years [9].

Cold Atmospheric Plasma (CAP) is a non‐invasive form of energy containing neutral particles, electrons, ions, and reactive oxygen/nitrogen species (RONS) in the form of ionized gas. The application known as the ‘plasma shower’ is one example of the use of CAP technology in dermatology. In the in vitro study, Daeschlein et al. [10]. demonstrated that atmospheric plasma exhibits potent antibacterial activity against relevant pathogens even under conditions simulating a wound environment; this points to the plasma's potential antimicrobial effect on C. acnes. Furthermore, plasma's anti‐inflammatory and tissue regeneration‐promoting properties make it a versatile treatment option that targets several key mechanisms in acne pathogenesis simultaneously. Experimental and clinical studies have shown that plasma application provides rapid regression of inflammatory acne lesions and that the treatment is well tolerated [11, 12].

On the other hand, laser systems have focused particularly on the vascular and pigmentary components in acne treatment. A pilot study by Yoon et al. demonstrated that long‐pulsed 595‐nm pulsed‐dye laser treatment improved acne erythema [13]. This and similar studies are based on the principle that laser energy is strongly absorbed by oxyhaemoglobin, targeting microvessels. A randomized controlled trial using pulsed‐dye laser (PDL) suggested that the 585 nm wavelength creates a photodynamic effect on C. acnes and can also modulate the local immune response by creating non‐lethal heating in the dermal perivascular area, thereby reducing the inflammatory response [14]. 585 nm lasers operating in Q‐Switch mode have shorter pulse durations compared to PDLs of similar wavelengths and, with these characteristics, show promising results, particularly in the treatment of post‐inflammatory erythema (PIE) and early‐stage acne scars [15, 16]. However, the number of studies directly evaluating the efficacy of Q‐Switch 585 nm lasers on acne vulgaris and comparing them with other physical modalities, such as cold atmospheric plasma, is quite limited.

The aim of this study is to comparatively evaluate the clinical efficacy, safety profiles, and treatment response processes of two distinct physical treatment methods that are gaining increasing popularity in the treatment of mild to moderate acne vulgaris: Cold atmospheric plasma shower and 585 nm Q‐Switch laser. The study aims to shed light on the clinical applications of both methods based on their different targets in acne pathogenesis and to provide clinicians with evidence‐based guidance for treatment selection.

## 2 Materials and Methods

### 2.1 Study Design and Power Analysis

This study was designed as a prospective, comparative, open‐label, non‐randomized clinical study conducted at the Dermatology Outpatient Clinic of Inonu University Faculty of Medicine between September 2023 and March 2025. The reporting of this study adheres to the STROBE (Strengthening the Reporting of Observational Studies in Epidemiology) guidelines for observational studies [17].

Prior to patient enrollment, a power analysis was performed using G*Power 3.1 software to determine the required sample size for repeated measures ANOVA (with interaction). The analysis indicated that a sample size of 46 participants would be sufficient to detect a moderate effect size (f = 0.25) with 80% power (1‐β) and a significance level of 0.05 (α). A total of 47 patients were enrolled to account for potential dropouts.

### 2.2 Study Population

The study protocol was approved by the Clinical Research Ethics Committee of Inonu University Faculty of Medicine (Decision No: 2025/7555). All participants provided written informed consent prior to enrollment.

A total of 47 patients aged 18 years and older, diagnosed with mild to moderate acne vulgaris, were prospectively enrolled in the study. Patients were allocated to either the plasma shower group or the 585 nm Q‐switch laser group based on clinical decision and patient preference. Only patients who completed the two treatment sessions and all follow‐up visits (week 2 and week 4) were included in the final analysis.

### 2.3 Exclusion Criteria

Patients with any of the following conditions were excluded from the study:

Dermatological diseases such as psoriasis, vitiligo, or lichen planus showing a positive Koebner phenomenon.

Individuals unable to cooperate due to psychiatric issues.

Unrealistic treatment expectations.

Known photosensitivity.

Presence or suspicion of malignant skin lesions.

History of active erosion, ulceration, or keloids.

Recent history of sunburn or tanned skin.

Receiving systemic or topical acne treatment before or during the study period.

Additionally, patients with incomplete data regarding the treatment process were excluded from the analysis.

### 2.4 Clinical Assessment and Data Collection

The primary measurement of acne severity was performed using the Global Acne Grading System (GAGS), which has proven validity and reliability [18]. This measurement enabled the objective and quantitative assessment of treatment efficacy. According to this system, scores between 1 and 18 were classified as ‘mild’ acne, while scores between 19 and 30 were classified as ‘moderate’ acne. The GAGS scores of all patients were systematically recorded at three different time points as part of the prospective study protocol: At baseline (week 0, prior to the first treatment), at week 2 (two weeks after the first session), and at week 4 (four weeks after the second session). Assessment of post‐inflammatory erythema (PIE) was performed clinically by two independent dermatologists based on standardized digital photographs taken under consistent lighting conditions without the use of objective instrumental measurements.

### 2.5 Treatment Protocols

Patients were divided into two groups according to the treatment applied:

**Plasma Shower Group (*n* = 24):** In the application, the Plasma CT device was used at maximum power and in continuous mode with the Plasma Shower head. The procedure was performed by first scanning the entire face and then applying additional treatments of approximately 5 min each to the areas with the highest concentration of lesions (right/left cheek, forehead).

**585 nm Q‐Switch Laser Group (*n* = 23):** The Lutronic Spectra Q‐Switch laser system and Gold Toning head were used for treatment at a wavelength of 585 nm. The laser parameters were set to a pulse rate of 5 Hz, a spot size of 5 mm, and an energy density (fluence) of 0.50 J/cm^2^. After a general scan of the entire face, 3–4 additional pulses were applied to the inflammatory lesions.

A total of two treatment sessions were performed in both groups, with a two‐week interval between sessions. This protocol was determined to preserve the skin barrier and minimize the risk of side effects in low‐energy laser and plasma applications and is consistent with the session intervals used in similar laser studies [15].

### 2.6 Statistical Analysis

Statistical analyses were performed using IBM SPSS Statistics 29.0 (MacOS) software. The normality of quantitative data was assessed using the Shapiro–Wilk test. The independent samples *t*‐test was used to compare basic differences between groups.

To analyze the effect of treatment over time, Repeated Measures ANOVA was applied; when the sphericity assumption was violated, the Greenhouse–Geisser correction was applied. Where significant, pairwise comparisons were examined using Bonferroni‐corrected tests. An independent samples *t*‐test was used to compare the score changes between the two treatment groups at baseline and week 4. Factors affecting the baseline‐week 4 score difference were evaluated using Multiple Linear Regression Analysis. In all analyses, the statistical significance level was set at *p* < 0.05.

## 3 Results

This study included a total of 47 patients, comprising 24 patients who underwent plasma shower therapy and 23 patients who underwent 585 nm Q‐Switch laser therapy. The demographic characteristics of both groups are compared in Table 1, and no statistically significant differences were found between the groups in terms of age, gender distribution, and Fitzpatrick skin type (*p* > 0.05 for all). The mean age of the plasma shower group was 23.1 ± 4.4 years, while that of the Q‐Switch laser group was 21.7 ± 1.9 years.

**TABLE 1:** Demographic and baseline clinical characteristics of the patient groups.

Characteristic	Plasma shower (*n* = 24)	Q‐Switch laser (*n* = 23)	*p*
Age (years), mean ± SD	23.1 ± 4.4	21.7 ± 1.9	0.368*
**Gender, *n* (%)**			
Male	6 (25.0)	4 (17.4)	0.724**
Female	18 (75.0)	19 (82.6)	
**Fitzpatrick Skin Type, *n***			
I	0	3	0.076***
II	6	8	
III	12	11	
IV	6	1	
Initial GAGS Score, Mean ± SD	16.3 ± 4.0	16.3 ± 2.8	0.864*

Abbreviations: GAGS, Global Acne Grading System; SD, Standard Deviation.

* Mann–Whitney U test.

** Fisher's Exact Test.

*** Pearson Chi‐Square test.

### 3.1 Changes in Treatment Efficacy Over Time

**FIGURE 1:**
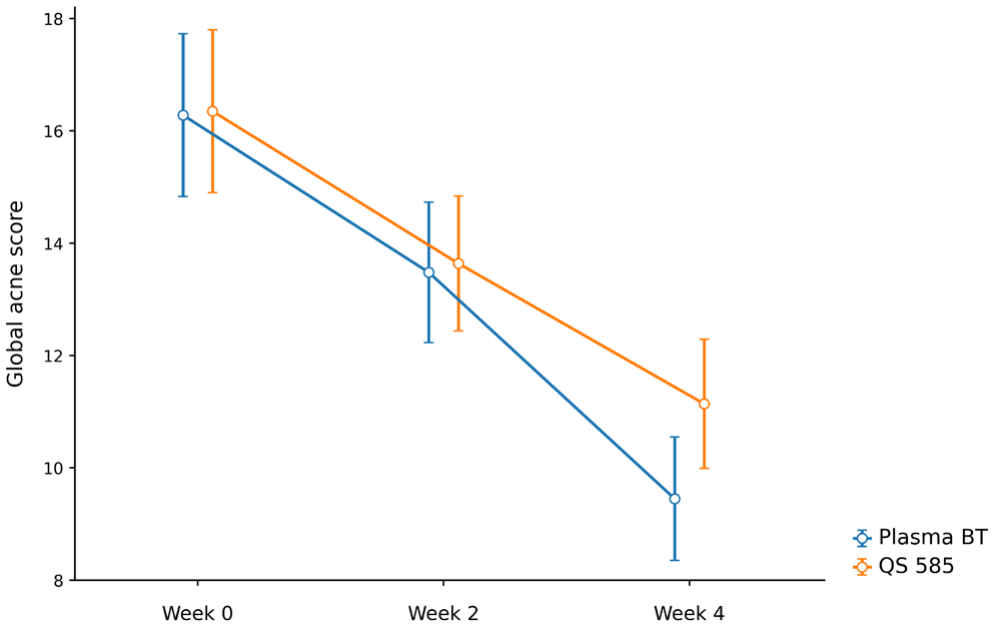
Time‐dependent change in GAGS scores in both treatment groups.

Changes in GAGS scores showed a statistically significant decrease at all follow‐up points in both treatment groups (Figure 1). In the plasma shower group, GAGS scores decreased from 16.3 ± 4.0 at baseline to 13.4 ± 3.4 at week 2 and 9.4 ± 3.0 at week 4. In the Q‐Switch 585 nm laser group, these values were recorded as 16.3 ± 2.8, 13.8 ± 2.2, and 11.0 ± 2.2, respectively.

Repeated measures ANOVA analysis showed that the time factor was statistically significant (*p* < 0.001). More importantly, the interaction between time and treatment group was found to be statistically significant (*p* = 0.003). This finding indicated that the two treatment methods exhibited different recovery curves over time. Bonferroni‐corrected comparisons confirmed that the differences in GAGS scores between all time points were significant in both groups (*p* < 0.001 for all) (Table 2).

**TABLE 2:** Results of the Repeated‐Measures ANOVA for GAGS scores.

Source of variation	Sum of squares (SS)	Degrees of freedom (df)	F	*p*	Partial eta squared (η^2^p)
Time	863.0	2	367.77	**< 0.001**	0.891
Time × Treatment Group	14.5	2	6.16	**0.003**	0.120
Error (Within)	105.6	90			

*Note:* Significant *p*‐values are indicated in bold.

Abbreviations: GAGS, Global Acne Grading System; SS, Sum of Squares.

### 3.2 Intergroup Efficacy Comparison

The change in GAGS scores between baseline and week 4, the primary indicator of treatment efficacy, was significantly higher in the plasma shower group compared to the Q‐Switch laser group. This difference was found to be statistically significant by an independent samples *t*‐test (*p* = 0.007). The effect size was calculated as Cohen's d = 0.82, indicating a high effect size (Table 3).

**TABLE 3:** Comparison of GAGS scores change from baseline to week 4 between treatment groups.

Statistical test	Result	*p*
Independent *t*‐test	t(45) = 2.80	**0.007**
Mean Difference (Plasma—Laser)	1.49 (95% CI: 0.42 to 2.56)	
Effect Size (Cohen's d)	0.82 (95% CI: 0.22 to 1.41)	

*Note:* Boldface indicates statistical significance (*p* < 0.05).

Abbreviations: CI, Confidence Interval; GAGS, Global Acne Grading System.

The percentage improvement rates also supported this superiority. While an average decrease of 42.5% ± 9.3% was observed in the plasma shower group at week 4 compared to the baseline score, the average decrease in the Q‐Switch laser group was 32.2% ± 8.4% (Table 4).

**TABLE 4:** Percentage improvement rates in GAGS scores from baseline to Week 4.

Treatment group	*n*	Mean ± SD (%)	Median (%)	Range (%)
Plasma Shower	24	42.5 ± 9.39	41.7	26.7–70.0
QS 585 nm Laser	23	32.2 ± 8.41	33.3	16.7–47.1

Abbreviations: QS, Q‐Switch; SD, Standard Deviation.

### 3.3 Analysis of Factors Affecting Clinical Response

In the multiple linear regression analysis conducted to examine the factors affecting the difference in GAGS scores between baseline and week 4 (amount of improvement), the model was found to be generally significant (*p* < 0.001). The analysis revealed that a higher baseline GAGS score (β = 0.35, *p* < 0.001) and the application of plasma shower therapy (β = −1.66, *p* < 0.001) were statistically significant and independent predictors of the amount of improvement. In contrast, age, gender, and Fitzpatrick skin type were not found to have a significant effect on treatment response (Table 5).

**TABLE 5:** Multiple linear regression analysis of factors affecting the change in global acne score (baseline to Week 4).

Independent variable	Regression coefficient (b)	Standard error	Standardized coefficient (beta)	*t*	*p* *
Constant (Intercept)	1.265	2.042	—	0.620	0.539
Age	−0.001	0.064	−0.002	−0.010	0.992
Baseline GAGS Score	0.350	0.065	0.589	5.395	**< 0.001**
Gender (Ref: Female)	−0.826	0.529	−0.173	−1.560	0.127
Treatment (Ref: QS Laser)	−1.656	0.447	−0.405	−3.701	**< 0.001**
Fitzpatrick Type 4 (Ref: Type 3)	0.191	0.650	0.041	0.294	0.770
Fitzpatrick Type 2 (Ref: Type 3)	0.084	0.505	0.020	0.166	0.869
Fitzpatrick Type 1 (Ref: Type 3)	0.779	0.910	0.102	0.856	0.39

Abbreviation: GAGS, Global Acne Grading System.

* The dependent variable is the difference between Baseline and Week 4 GAGS scores. Significant *p*‐values are indicated in bold.

### 3.4 Visual Clinical Improvement

**FIGURE 2:**
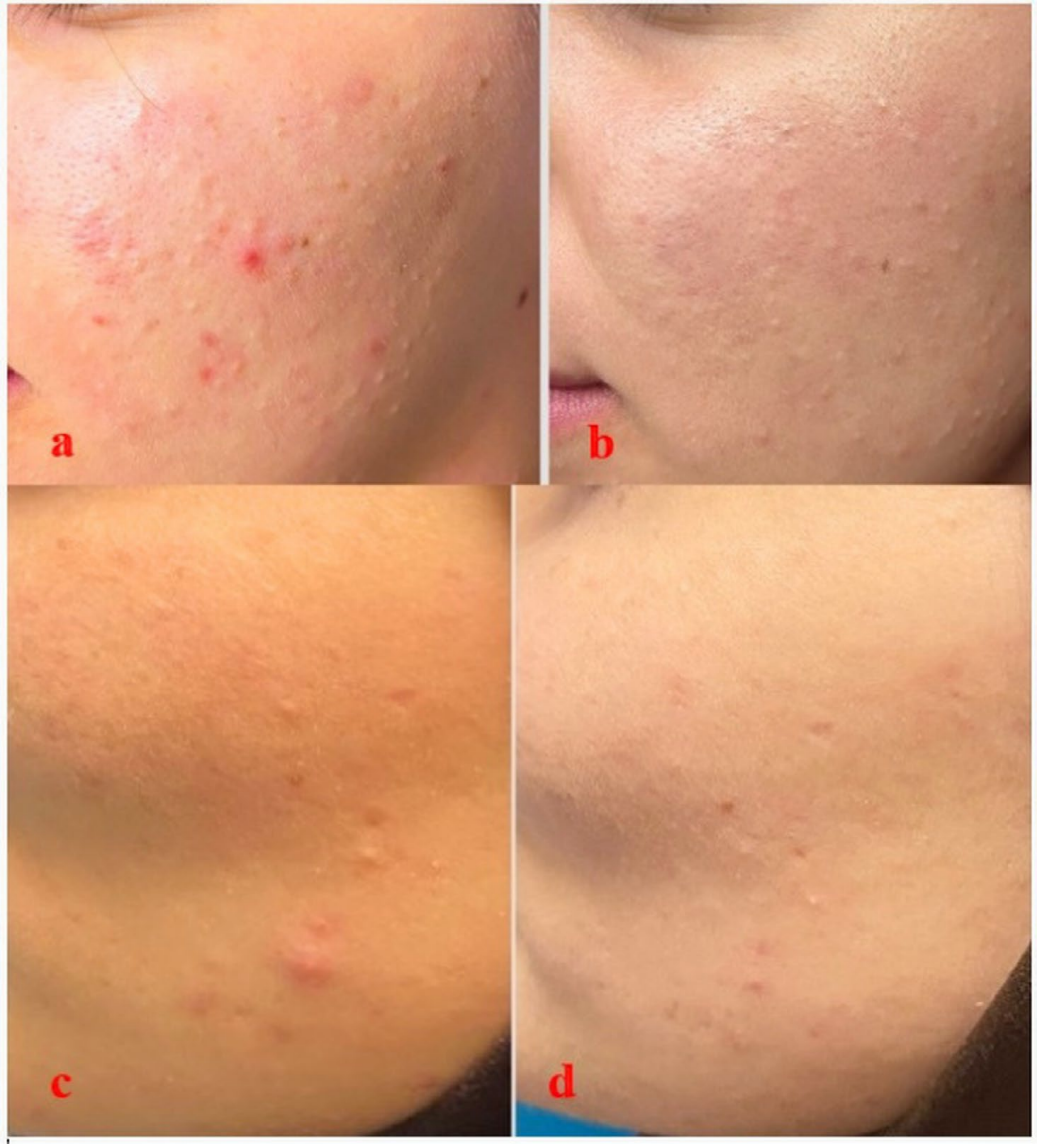
Acne on the left cheek before plasma shower treatment (a, c) and at week 4 (b, d).

**FIGURE 3:**
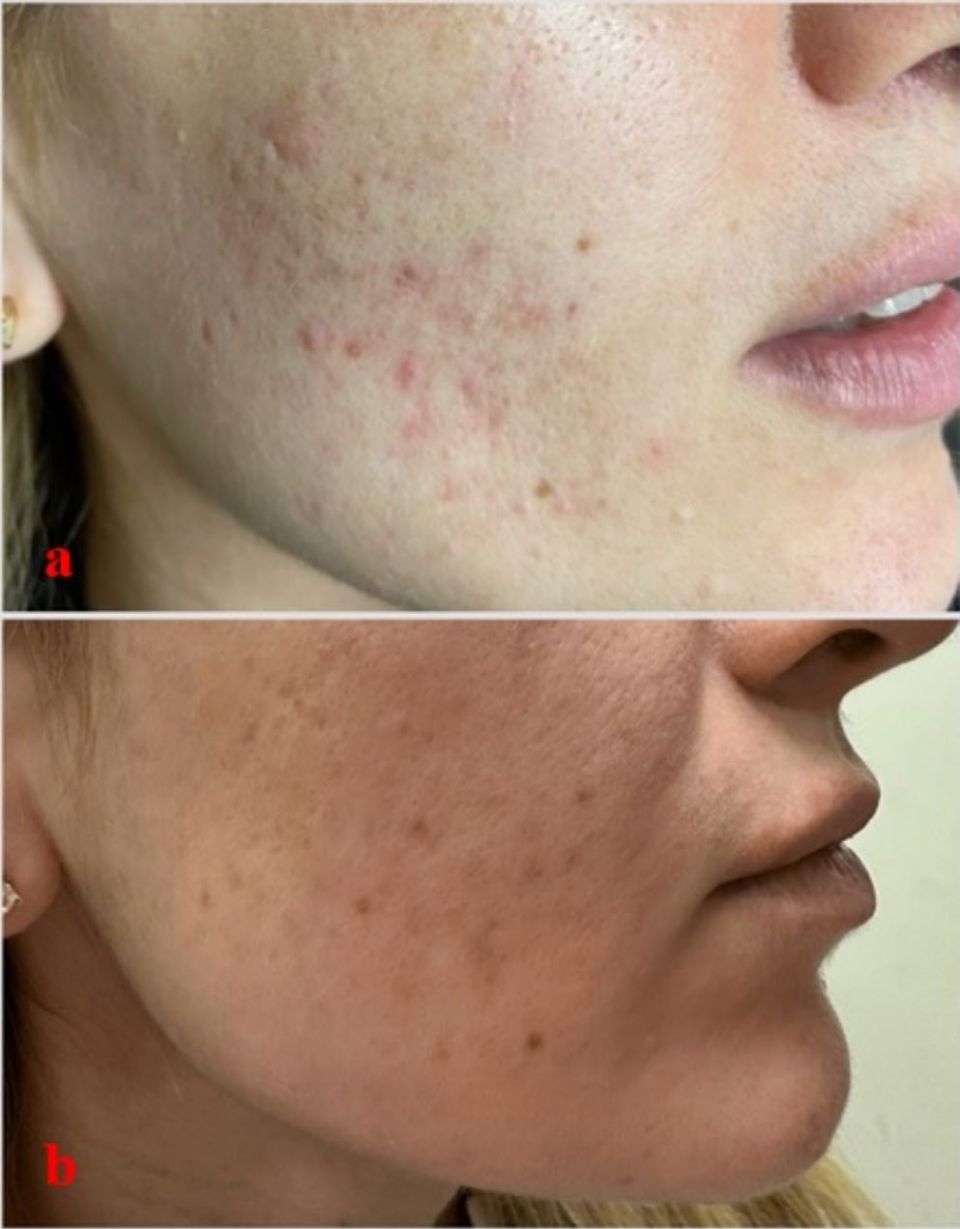
Acne on the right cheek before plasma shower treatment (a) and at week 4 (b).

**FIGURE 4:**
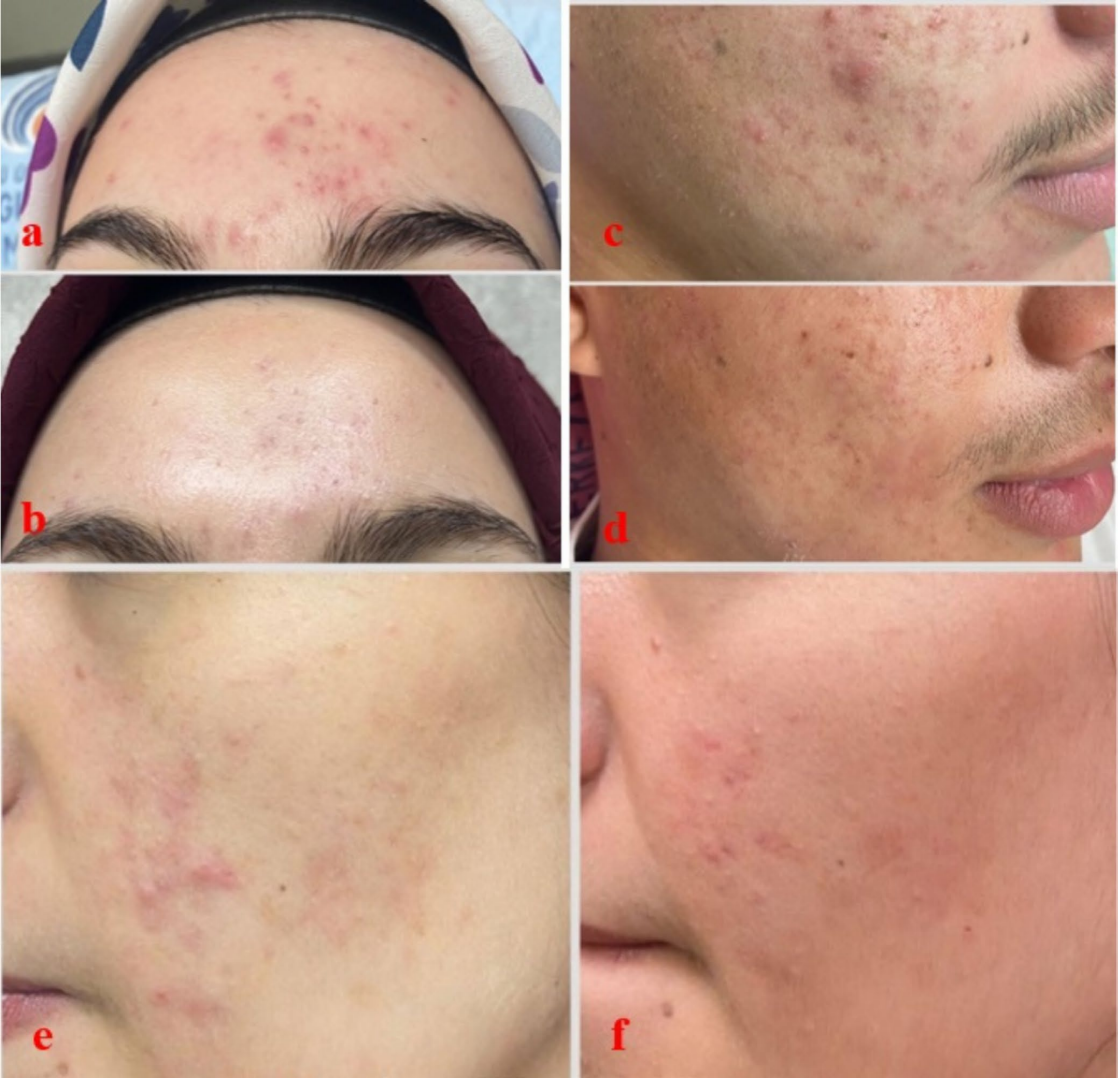
Acne before Q‐Switch 585 nm laser treatment (a, c, e) and at week 4 (b, d, f).

The clinical efficacy of the treatments is also supported by patient photographs. In patients undergoing plasma shower therapy, a marked reduction in inflammatory papules and pustules was observed in both the left and right cheek areas by the fourth week (Figure 2 and Figure 3). In the group receiving 585 nm Q‐Switch laser therapy, a notable lightening and reduction in redness of post‐inflammatory erythema (PIE) on the forehead and cheeks was noted (Figure 4). These visual findings are consistent with the statistical results and visually confirm the different clinical components targeted by each treatment (active inflammation and vascular erythema, respectively).

### 3.5 Safety and Tolerability

Both treatment methods were generally well tolerated. In the plasma shower group, temporary and mild erythema and a stinging sensation were reported after application, but these side effects resolved spontaneously within a few hours. In the Q‐Switch laser group, no significant adverse events were observed after treatment other than mild oedema. No patient experienced serious side effects, permanent pigment changes, or scarring.

## 4 Discussion

This study comparatively evaluated the efficacy and safety profiles of cold atmospheric plasma (Plasma Shower) and 585 nm Q‐Switch laser in the treatment of mild to moderate acne vulgaris. The findings revealed that both methods provided a significant improvement in global acne scores over a four‐week period; however, Plasma Shower treatment demonstrated a statistically significant superiority in terms of both the rate of improvement and the level of efficacy.

In this study, the average 42.5% reduction in GAGS scores observed in the Plasma Shower group supports the efficacy of this method in acne treatment. This result is consistent with previous findings in the literature. For example, Chutsirimongkol et al. [19]. reported that plasma application resulted in a significant reduction in inflammatory lesions. The efficacy of Plasma Shower is likely due to its multifaceted mechanism of action. An in vitro study by Daeschlein et al. [10]. confirmed the antibacterial effect of atmospheric plasma; Rutkowski et al. [12]. emphasized that it stimulates tissue regeneration and produces anti‐inflammatory effects via reactive oxygen and nitrogen species (RONS). Therefore, it can be considered that Plasma Shower simultaneously targets multiple factors in acne pathogenesis, such as suppressing C. acnes colonization, modulating sebum production, and reducing the inflammatory cascade. Furthermore, a prospective study by Karrer et al. [11]. reporting a reduction of up to 52% in inflammatory lesions on the side treated with plasma therapy supports our findings.

On the other hand, the average 32.2% improvement observed in the 585 nm Q‐Switch laser group suggests that this modality is also effective, but its primary area of effect may be different. In our study, although the absolute improvement of the Q‐Switch laser in inflammatory lesions was lower compared to Plasma Shower, our clinical observation is that this method has a more pronounced effect, particularly on post‐inflammatory erythema (PIE). This finding is in complete agreement with the literature. Panchaprateep and Munavalli [15] reported that the low‐energy 585 nm Q‐Switch laser provided significant improvement in PIE and high patient satisfaction. Similarly, Wang et al. [20]. also reported that the 585 nm Q‐Switch laser had a significant effect on PIE, but its effect on inflammatory lesions was limited. This selective effect of the Q‐Switch laser can be explained by the strong absorption of the 585 nm wavelength by oxyhaemoglobin, thereby targeting microvessels. A study by Seaton et al. [14]. using a Pulsed‐Dye Laser suggested that this wavelength could also provide photodynamic effects and immunomodulation on C. acnes. However, the much shorter pulse durations of the Q‐Switch mode may be more effective for vascular targeting rather than suppressing the inflammatory process.

Both treatment methods were well tolerated by patients, with no serious or persistent side effects observed. The transient erythema and stinging sensation seen with Plasma Shower and the mild oedema seen with Q‐Switch laser are expected reactions consistent with the reported literature, resolving spontaneously within a short period [11, 15]. This safety profile suggests that both methods can be used safely in patients who cannot tolerate topical agents, have contraindications to systemic treatment, or are resistant to drug therapy.

Limitations of our study include the relatively small sample size, short follow‐up period, single‐center design, and the non‐randomized allocation of patients, which may introduce selection bias. Future randomized controlled trials with longer follow‐up are warranted to confirm these findings. Furthermore, objective measurements of PIE (e.g., spectrophotometry) were not used, and findings in this regard are based on clinical observation.

In conclusion, based on the findings of this study, for the treatment of mild to moderate acne vulgaris:

- Cold atmospheric plasma (Plasma Shower) stands out as the first‐line physical therapy option in cases where active inflammation is prominent and inflammatory lesions need to be rapidly reduced.
- The 585 nm Q‐Switch laser should be considered an effective and safe adjunctive modality for the resolution of post‐inflammatory erythema that develops or becomes prominent after the treatment course.
- Both methods are valuable options in clinical practice, either alone or in combination protocols, with low side effect profiles and good patient compliance, empowering the clinician.
- In the future, there is a need for randomized controlled trials examining broader patient groups, longer follow‐up periods, objective measurement methods, and the synergistic effects of combining plasma and laser treatments.

During the preparation of this work, the authors used ChatGPT AI for language editing purposes. After using this tool/service, the authors reviewed and edited the content as needed and take full responsibility for the content of the published article.

## Author Contributions

Celal Alperen Bakircioglu: Supervision, conceptualization, visualization, writing – original draft, data analysis, literature review. Dursun Turkmen: Supervision, conceptualization, visualization, writing – original draft, data analysis, literature review. Nihal Altunisik: Data analysis, data curation, visualization. Zekiye Kanat: Data analysis, literature review. Serpil Sener: Data analysis, manuscript editing. Cemil Colak: Supervision, conceptualization, visualization, writing – original draft.

## Funding

The study was supported by the Scientific Research Projects Coordination Unit of Inonu University with the project code TSG‐2022‐3111. This study was approved by the Clinical Research Ethics Committee of Inonu University Medical Faculty (2025/7555).

## Conflicts of Interest

The authors declare no conflicts of interest.

## Data Availability

The data that support the findings of this study are available on request from the corresponding author. The data are not publicly available due to privacy or ethical restrictions.
